# The apoptotic effect of garlic *(Allium sativum)* derived SEVs on different types of cancer cell lines in vitro

**DOI:** 10.55730/1300-0152.2694

**Published:** 2024-05-28

**Authors:** Naz ÜNSAL, Polen KOÇAK DENİZCİ, Hazal YILMAZ, Fikrettin ŞAHİN, Merve YILDIRIM CANPOLAT

**Affiliations:** 1 Department of Genetics and Bioengineering, Faculty of Engineering and Architecture, Yeditepe University, İstanbul, Turkiye; 2 Department of Biomedical Engineering, Faculty of Engineering and Natural Sciences, İstinye University, İstanbul, Turkiye

**Keywords:** Plant-derived extracellular vesicles, apoptosis, cancer

## Abstract

**Background/aim:**

Small extracellular vesicles (SEVs) are known to have an impact on the physiological conditions of target cells, are a critical component of cell-to-cell communication, and have been implicated in a variety of diseases. Although it has been proposed that edible plant-derived nanoparticles have an effect on communication with mammalian cells, the influence of these nanoparticles on cancer cell development has yet to be explored.

**Materials and methods:**

In order to characterize small extracellular vesicles obtained from garlic, specific SEV surface markers, antibodies, and size detections were identified using scanning electron microscopy and nanoparticle tracking analysis. Human hepatoma (Hep3B), human neuroblastoma (SH-SY5Y), human pancreatic adenocarcinoma (Panc-1a), human glioblastoma (U87), prostate cancer (PC-3), and human umbilical vein endothelial (HUVEC) cell lines were treated with garlic SEVs to examine their anticancer properties.

**Results:**

Annexin V FITC/PI staining for apoptosis, mRNA, and protein expression levels via RT-PCR and ELISA indicated that garlic SEVs triggered apoptosis by activating the intrinsic pathway. Our findings support the idea that SEVs produced from garlic may trigger apoptotic cell death in cancer cells while having no effect on healthy cells.

**Conclusion:**

It was discovered that plant SEVs had anti-cancer effects by activating caspase-mediated apoptosis.

## 1. Introduction

Cancer is the uncontrolled division and proliferation of cells that have been altered by certain effects in an organism. Cancer has become an increasing problem worldwide, and numerous methods have been improved for the development of cancer therapy strategies (Nenclares and Harrington, 2020). To exemplify, current methods such as radiotherapy and chemotherapy could have side effects such as damaging healthy cells as well as cancer cells. Therefore, last scientific studies have focused on green medicine, plant-based therapies and strategies (Li et al., 2017).

Prostate cancer has become an important health issue since it is the second most common type of cancer seen in men all over the world, and according to 2014 data, the incidence of prostate cancer in men is 32.9 per 100,000 in Türkiye (Culp et al., 2020). According to 2012 data, men in the United States have a probability of one in seven of developing lifelong prostate cancer (Bilgili et al., 2019). When pancreatic cancer is mentioned, adenocarcinomas first come to mind, and these pancreatic adenocarcinomas have a poor prognosis from the early stages and do not have a curative effect, even after intensive treatments. Although the incidence of pancreatic cancer ranks 11th in the United States, it ranks 4th in cancer-related deaths in men and women after other types of cancer (Haidinger et al., 2021). According to 2013 data, the incidence of pancreatic cancer in men is 6.3 per 100,000 and in women 3.6 per 100,000 in Türkiye (Doğan et al., 2020).

Neuroblastoma is a type of malignant tumor that is frequently observed in early childhood and originates from the sympathetic nervous system. Neuroblastomas constitute almost 7% of all cancers in children and adolescents, and these are the most common solid tumors after central nervous system tumors (Van Heerden et al., 2021). According to the German Children’s Cancer Database Mainz, approximately 130 children under the age of 14 develop neuroblastomas each year in Germany; therefore, approximately one in every 100,000 children under the age of 15 develops this disease each year.

Glioblastoma is the most common type of brain tumor; it can occur at any age; however, it is particularly prevalent between the ages of 45 and 75. The incidence of glioblastoma was found to be 3.19 per 100,000 per year (Chen et al., 2021). Malignant tumors originating from the liver’s own cells are called primary liver cancer, and this type of cancer is called hepatocellular (liver cell) carcinoma because it originates from the liver’s own cells. It is the fifth most common type of cancer, and its incidence is 20–200 per 100,000. In addition, this cancer type ranks third among the deadliest tumors (Petrick J et al., 2020). As a result of these, in this study, we aimed to develop a plant-derived exosome-like extracellular vesicles-based therapy strategy, which is a new approach in cancer treatment.

Exosomes are nanosized vesicles that play a role in cell-cell interaction and communication, consisting of RNA and protein released by cells. These nanoparticles play an important role in the transmission of hereditary materials through intracellular communication and in multiple biological events, such as the regulation of responses in the immune system (Von Schulze and Deng, 2020). Extracellular vesicles obtained from plants are known to exhibit similar properties to mammalian exosomes (Kim et al., 2022). The effects of extracellular vesicles from many different plants have still not been investigated enough, including the effect on cancer cells, wound healing, and drug delivery (Zhang et al., 2020).

Garlic *(Allium sativum)* is an important plant for the nutrition of people worldwide. Researches show that the consumption of garlic could protect against cancer, reduce blood sugar, and provide many benefits to the body. Garlic has been frequently used in the treatment of diseases such as typhus, influenza, dysentery and cholera. With developing studies, garlic is now being used in the development of new therapy strategies (Batiha et al., 2020). This study shows that extracellular vesicles of garlic *(Allium sativum)* can be an alternative approach for the treatment of cancer. In this study, the apoptotic effect of garlic exosomes like small extracellular vesicles (garlic SEVs), characterized with various methods, was investigated using different techniques such as cytotoxicity assay, RT-PCR, Annexin V FITC/PI, TUNEL assay, and ELISA protein assay.

## 2. Materials and methods

### 2.1. Cell culture and treatment with garlic derived SEVs

Human hepatoma cell line Hep3B (ATCC HB-8064), human neuroblastoma cell line SH-SY5Y(ATCC CRL-226), human pancreatic adenocarcinoma cell line Panc-1 (ATCC CRL-1469), human umbilical vein endothelial cell line HUVEC (ATCC CRL-1730), human glioblastoma cell line U87 (ATCC HTB-14) and prostate cancer cell line PC-3 (ATCC CRL-1435) were obtained from the American Type Culture Collection. These cell lines were cultured with complete Dulbecco’s modified Eagle medium (DMEM) High (Invitrogen) supplemented with 10% (v/v) FBS (fetal bovine serum) and 100 units/mL Penicillin/Streptomycin/Amphotericin (1%) antibiotics were placed in tissue culture flasks and incubated in NuAire NU-5510/E/G incubator at 37 °C in a highly humidified atmosphere (RH 80%) with 5% CO_2_ rate and 95% (v/v) air. Treatment with garlic exosome-like extracellular vesicles was performed at a concentration of 60 μg/mL.

### 2.2. Garlic derived SEV isolation and characterization

#### 2.2.1. Garlic derived SEV isolation

Garlic *(Allium sativum)* was obtained from the local market. First, garlic was blended with distilled water. Then garlic juices were centrifuged at 10,000 g for 40 min. The supernatant was collected and filtered by 0.45 to remove large particles. Then the filtered solution was mixed with DEX-PEG solution (30 g/L dextran, 70 g/L polyethylene glycol) in a 1:1 volume ratio. These solutions were centrifuged at 1000 g for 10 min. After centrifugation, 80% from the upper phase of solution was discarded. The washing solution was prepared by mixing DEX-PEG and distilled water with the 1:1 volume ratio. The washing solutions were also centrifuged at 1000 g for 10 min. Then 80% from the upper phase of the washing solution was added to the extracellular vesicle solution. The new solution was centrifuged at 1000 g for 10 min. The upper phase was discarded, and the bottom phase was filtered by 0.22 μm.

#### 2.2.2. SEV characterization with flow cytometer

Isolated garlic exosome-like extracellular vesicle samples were mixed with 1 μL of CD9, CD63, and HSP70 antibodies. Samples were incubated at room temperature in a dark environment, and measurements were performed using FACSCalibur flow cytometry.

#### 2.2.3. SEV characterization with nanoparticle tracking analysis

Isolated garlic exosome-like extracellular vesicles were loaded to NanoSight by using a syringe. Exosome images were taken by adjusting threshold settings. The number of particles was also measured.

#### 2.2.4. SEV characterization by scanning electron microscope

Garlic exosome-like extracellular vesicle samples were dissolved in 1 mL PBS. Two drops of this solution were dropped on the slide and air-dried. SEV images were obtained under a scanning electron microscopy.

### 2.3. Cytotoxicity assay

All cell lines were seeded into 96-well plates at a concentration of 5 × 103 cell/well and after one day cells were treated with different concentrations of garlic exosomes. After 24 h, these cell lines which were treated with the exosomes and the medium were discarded from the wells. 100 μL of glucose-PBS was mixed with 10 μL of MTS reagent for one well and the total amount was calculated for each well. This solution was transferred into each well of the plates and plates were incubated at 37 °C for 1 h. After the incubation, the plate was read at 490 nm in Elisa plate reader. In addition, this process was done for 48 h and 72 h of treatment.

### 2.4. TUNEL assay

All cell lines were seeded in 8-well chambers at a concentration of 5 × 103cell/well. Each cell line has two wells: one for control and one for treatment with garlic exosome. The day after, cells were treated with 60 μg/mL of garlic exosome. After 18 h, cells were fixed, and Click-iT Plus TUNEL assay for in situ apoptosis detection protocol was used in order to detect apoptotic cells with the dye DAPI for the nucleus. All images were obtained under a confocal microscope.

### 2.5. Real-time PCR

Bax, Bcl-2, Cas9, Cas3, and p53 primers were selected as apoptotic markers for detecting expression levels of these genes via real-time PCR analysis. Quanti-Tech SYBR Green PCR kits were applied.

### 2.6. ELISA

Medium of cells that were treated with 60 μg/mL SEVs for 48 h were collected into Eppendorf tubes. The removable 8-well strips included in the kit (Bio-Rad, USA) were labeled as much as the number of samples. Following the Human Caspase-3 ELISA Kit protocol, 100 μL of each standard and sample were added into labeled wells. The wells were then covered and incubated at room temperature for 2.5 h with gentle shaking. After incubation, the solution was discarded and washed four times with 1x wash solution. Next, 100 μL of 1x prepared biotinylated antibody was added to each well and incubated for 1 h at room temperature with gentle shaking. Then, the washing step was repeated. Subsequently, 100 μL of prepared Streptavidin solution was added to each labeled well and incubated for 45 min at room temperature with gentle shaking. After this step, the washing step was repeated. Next, 100 μL of TMB One-Step Substrate Reagent was transferred to each well and incubated for 30 min at room temperature with gentle shaking in the dark. Then, 50 μL of Stop solution was added to samples and the plate was read at 450 nm immediately.

### 2.7. Flow cytometry analysis

The apoptotic effect of garlic SEVs was examined using th Annexin V detection kit in accordance with the manufacture’s protocol. All cell lines that were seeded with 1 × 105cell/well onto a 6-well plate were treated with 60 μg/mL of garlic SEVs. After 24 h of incubation, the cells were harvested and incubated with Annexin V FITC and PI PE-conjugated antibodies. Detections were performed using FACSCalibur flow cytometry. Data analysis was carried out using the CellQuest Pro program.

## 3. Results

**Figure 1:**
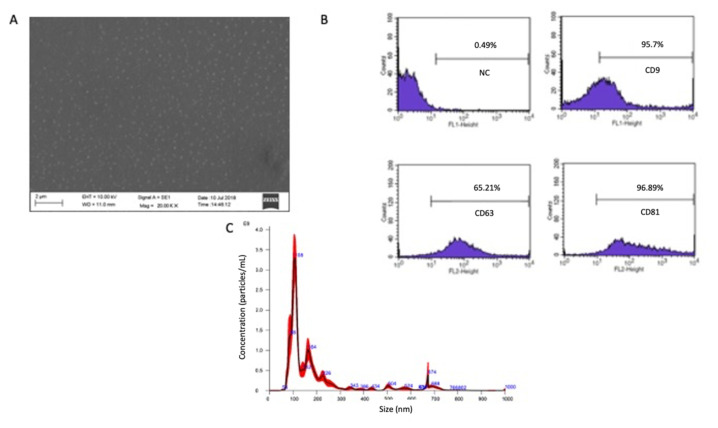
Scanning electron microscope and flow cytometry analysis of garlic SEVs isolated with ATPS method for the characterization. Scanning electron microscope image shows (A) various nanovesicles that are irregularly produced, with the scale bar indicating 2 m. Flow cytometry analysis of garlic SEVs (B) using the extracellular vesicle cell surface markers against CD9, CD63, and HSP70 antibodies. The size distribution of garlic SEV (C) nanoparticle tracking analysis (NTA) shows that the nanoparticles are between 50 and 160 nm in size.

The garlic SEVs were isolated using the ATPS method and were further analyzed using scanning electron microscopy and flow cytometry for characterization. Scanning electron microscope image reveals the presence of irregularly generated nanovesicles, as indicated by the scale bar measuring 2 m (Figure 1A). Garlic SEVs were analyzed using flow cytometry, specifically targeting the extracellular vesicle cell surface markers CD9, CD63, and HSP70 antibodies (Figure 1B). The nanoparticle tracking analysis (NTA) of garlic SEV reveals a size distribution ranging from 50 to 160 nm (Figure 1C).

**Figure 2:**
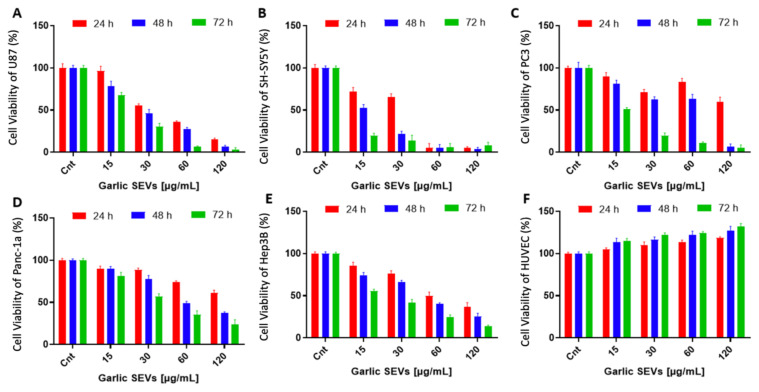
Analysis of the cell viability for human glioblastoma cell line, U87 (A), human neuroblastoma cell line, SH-SY5Y (B), prostate cancer cell line, PC-3 (C), human pancreatic adenocarcinoma cell line, Panc-1a (D), human hepatoma cell line, Hep3B (E), and human umbilical vein endothelial cell line, HUVEC (F) when treated with varying concentrations (15–120 μg/mL) of garlic exosome. Cells were sown in 96-well plates overnight and allowed to attach. The MTS experiment was carried out in DMEM supplemented with 10% FBS for 24, 48, and 72 h (37 °C, 5% CO_2_). The absorbance value of untreated control cells was compared to 100 to determine cell death. The absorbance value obtained from the negative control (NC), standard growth medium treated cells were assigned as 100% to assess the percentage of cell viability.

Effect of garlic SEVs on the cell survival of U-87, SH-SY5Y, PC-3, Panc-1a, HUVEC, and Hep3B cells. Garlic SEVs at concentrations up to 60 μg/mL had a significant cytotoxic effect on these cancer cell lines, reducing the viability of U87 (Figure 2A), SH-SY5Y (Figure 2B), PC-3 (Figure 2C), Panc-1a (Figure 2D), and Hep3B (Figure 2E) cells to 25–40% within 72 h. Significantly, the viability of normal HDF cells remained unaffected when the dosages of garlic SEVs were increased. Conversely, when HUVEC cells were subjected to garlic SEVs at moderate concentrations, their proliferation was considerably enhanced by up to 142% (Figure 2F).

**Figure 3:**
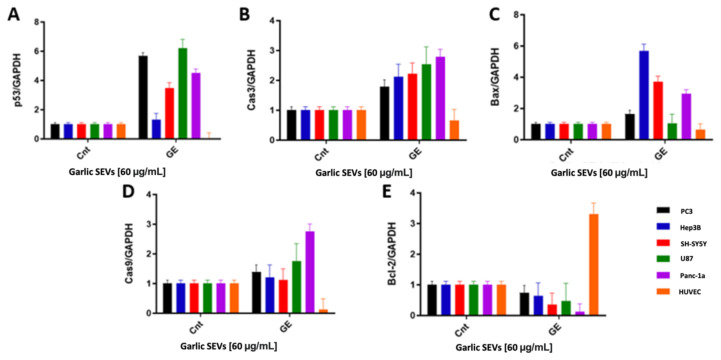
p53 (A), Cas3 (B), Bax (C), Cas9 (D), and Bcl-2 (E) expression levels in human glioblastoma cell line U87, human neuroblastoma cell line SH-SY5Y, prostate cancer cell line PC-3, human pancreatic adenocarcinoma cell line Panc-1a, human hepatoma cell line Hep3B, and human umbilical vein endothelium cell line HUVEC cells were quantified by RT-PCR. The housekeeping gene GAPDH was utilized as a negative control, and gene expression levels were standardized to it.

Using RT-PCR, we investigated the effect of 60 μg/mL garlic SEVs on pro- and antiapoptotic gene expression levels. The p53 (Figure 3A), Cas3 (Figure 3B), Bax (Figure 3C), Cas9 (Figure 3D), and Bcl-2 (Figure 3E) expression levels in U-87, SH-SY5Y, PC-3, Panc-1a, Hep3B, and HUVEC cell lines were analyzed. The antiapoptotic Bcl-2 gene expression level was significantly decreased down nearly 0.6-fold in PC-3, Hep3B, U87 cells, 0.4-fold in SH-SY5Y cells, and 0.2-fold in Panc-1a cells. Despite the fact that Bcl-2 gene expression was increased up to 3.5-fold in HUVEC cells, the difference was statistically significant (Figure 3E). When the expression levels of proapoptotic genes were examined in PC-3, it was observed that there was approximately a 1.5-fold increase detected in the expressions of the Bax and Cas9 genes. p53 gene expression levels increased by 6-fold and the Cas3 gene increased by 2-fold, respectively. For Hep3B cells, p53 and Cas9 genes expression levels increased by 1.5-fold, Cas3 gene expression levels increased by approximately 2-fold, and, lastly, Bax gene expression levels increased by 6-fold. According to the findings, the expression of proapoptotic genes p53 and Bax in the SH-SY5Y cell line increased by approximately 4-fold, while the expression of the Cas3 and Cas9 gene increased by nearly 2.5-fold and 1.3-fold, respectively. The Cas9, Cas3, and Bax gene expression levels increase by 1.8-, 2.7-, and 1.3-fold, respectively, for the U87 cell line, whereas the p53 expression levels increased by 6-fold. Cas3, Bax and Cas9 genes increase approximately by 3-fold, while p53 increased by 4-fold in the PC-3 cell line when the proapoptotic gene expression levels were assessed with RT PCR analysis.

**Figure 4:**
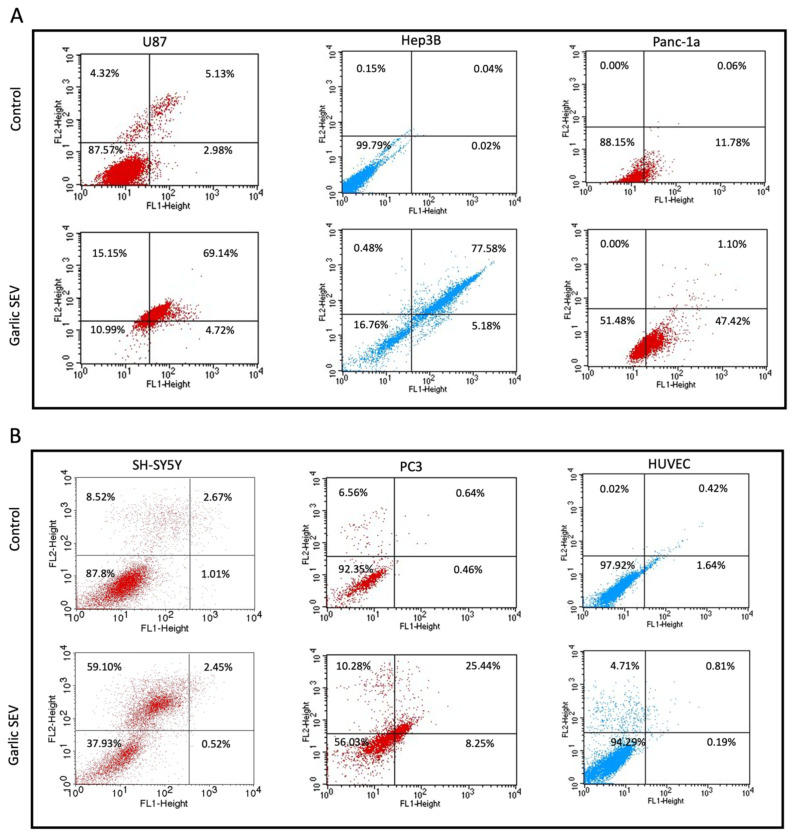
Annexin V/PI assay was applied on human glioblastoma U87, human hepatoma Hep3B, human pancreatic adenocarcinoma Panc-1a (Figure 4A), human neuroblastoma SH-SY5Y, prostate cancer PC-3, and human umbilical vein endothelial HUVEC cells (Figure 4B) in order to make detection of apoptosis following the treatment with 60 μg/mL garlic SEVs for 48 h. The cell cycle distribution analysis of HUVEC cells after the treatment of garlic SEVs (Figure 4C). The measurement of different stages was discussed. The data is expressed as the mean and standard deviation.

It was necessary to run Annexin V FITC/PI assays in order to validate the apoptotic effect of garlic SEVs on U-87, Panc-1a, PC-3, SH-SY5Y, and Hep3B cells in order to further examine the cell cycle arrest and cell death mechanisms of the cells. The treatment of garlic SEVs for U-87, Hep3B, and Panc-1a cells for 48 h revealed that apoptotic cell numbers were detected between 60% and 70% for late apoptosis rates. There was no substantial necrosis detected in U-87, Hep3B, and Panc-1a cells for 48-h treatment periods (Figure 4A). An increase in the rate of necrosis in SH-SY5Y and PC-3 cells was seen when they were treated with garlic SEVs at a concentration of 60 μg/mL for 48 h. After 48 h of treatment with SEVs, the apoptotic cell death ratio in PC-3 cells rose to 25.44%, indicating a significant increase in cell death (Figure 4B). In contrast, no substantial induction of apoptosis was detected in normal HUVEC cells, with the apoptotic cell ratio maintaining between 0.50% and 1%, throughout the 48-h treatment in response to garlic SEVs after exposure to the compounds (Figure 4B). For the cell cycle distribution analysis, there is no significant change detected for HUVEC cells (Figure 4C).

**Figure 5:**
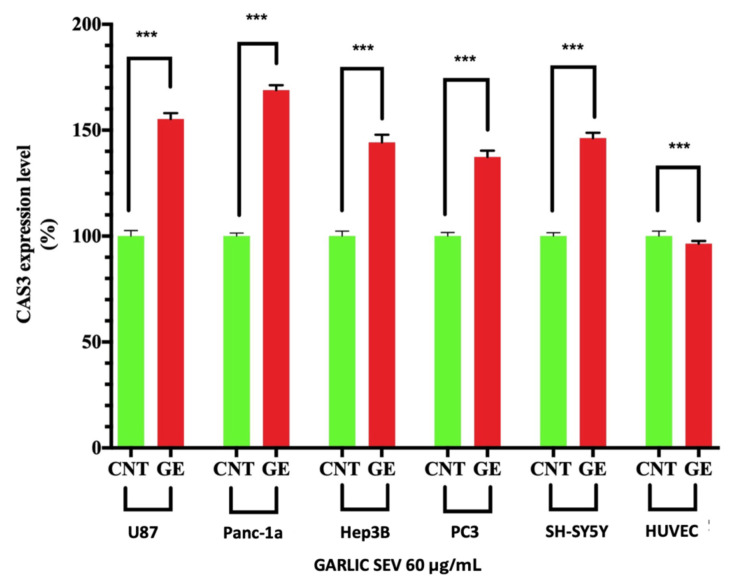
Effect of garlic SEVs on caspase-3 activity was determined by ELISA assay for U87, Panc-1a, Hep3B, PC3, SH, and HUVEC (*p ≤ 0.05, ** p ≤ 0.01, *** p ≤ 0.001, **** p ≤ 0.0001).

The effect of garlic SEVs on caspase-3 activity was measured by an ELISA test for active caspase-3. Figure 5 shows the effect of garlic SEVs on caspase-3 activation, as assessed by a commercially available caspase-3 ELISA kit. As shown in Figure 5, treatment of U87, Panc-1a, Hep3B, PC3, SH cells with garlic SEVs resulted in a statistically significant increase in caspase-3 activation when compared to cells that were not treated. There was no observed increasement in the healthy cell line HUVEC.

**Figure 6:**
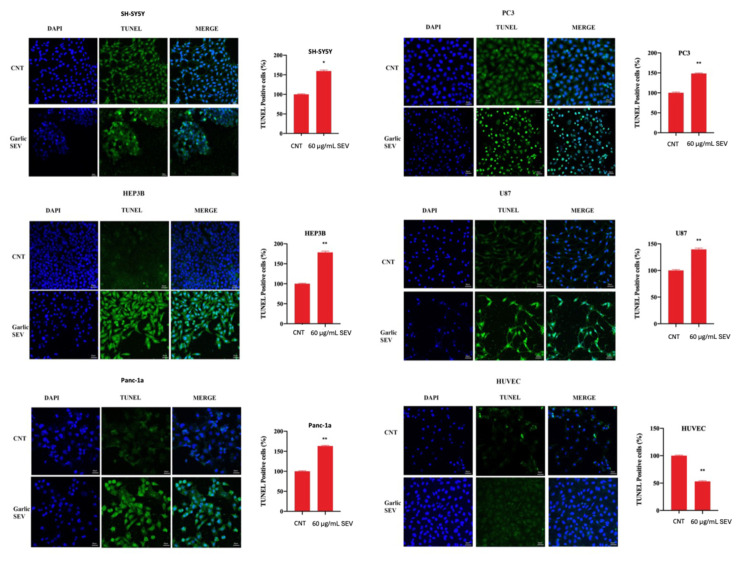
TUNEL assay of garlic SEVs-treated SH-SY5Y, Hep3B, Panc-1a, PC3, U87, and HUVEC cells. (* p ≤ 0.05, ** p ≤ 0.01, *** p ≤ 0.001, **** p ≤ 0.0001).

Furthermore, a TUNEL assay of garlic SEVs-treated cells was performed after 48 h of treatment to confirm the effect of garlic SEVs. In SH-SY5Y, Hep3B, Panc-1a, PC3, and U87, the mean florescence intensity of garlic SEVs-treated cells showed increased intensity compared with the untreated normal cells as shown in Figure 6.

## 4. Discussion

Exosomes, known as natural carriers, have high biodistribution and high stability in plasma, making them suitable candidates for therapeutic applications. It is possible to divide the therapeutic applications of exosomes into two categories: (i) using exosomes on their own or (ii) designing them to be carriers for an exogenic molecule. Herbal resources are seen as an important alternative in the diagnosis and treatment of many diseases (Tai et al., 2018). The plant group or species preferred in the use of these resources allow different diseases and applications according to the content they have (Horibe et al., 2018). Determining the chemical content of the plants also enables the determination of the disease or application in which this plant will be used (Zhang et al., 2020; Dad et al., 2021). Since plant exosome-like nanovesicles are naturally rich in bioactive lipids, proteins, ribonucleic acid, and other pharmacologically active compounds, they have distinct morphological and compositional properties that make them excellent natural nanocarriers (Fernandes et al., 2020). Recent research suggests that the components of these natural extracts, which contain SEVs, may be responsible for the previously reported effects of these extracts. Protein, lipid, and RNA content in plants with a wide size distribution (20–300 nm) approximate human SEVs (Rutter and Innes, 2018). Garlic’s anticarcinogenic potential has been proven in numerous studies employing various garlic preparations such as fresh garlic extract, aged garlic, garlic oil, and several organosulfur compounds produced from garlic (Thomson and Ali, 2005). Most studies indicated that it is a remarkable plant with multiple beneficial effects such as antimicrobial, antithrombotic, hypolipidemic, antiarthritic, hypoglycemic, and antitumor activity (Thomson and Ali, 2005).

Exosomes should be measured, at least three positive exosome protein markers should be present, and they should be seen using electron microscopy or NTA, according to MISEV guidelines (Gardiner et al., 2016; Théry et al., 2018). Flow cytometry was used to evaluate the presence of the tetraspanin proteins of CD9, CD63, and HSP70, which are well-known indicators of exosome membrane presence (Andreu and Yáñez-Mó, 2014). The size distribution of the exosome-like extracellular vesicles was quantified and acquired using nanoparticle tracking analysis. According to the literature, size distribution can range from 20 to 140 nm, as can the mean exosome size and distribution found by NTA and SEM. In this study, we demonstrated a strategy to isolate exosome-like nanovesicles from garlic. To investigate if garlic SEVs have an anticancer effect, we evaluated the effects of garlic SEVs treatment on the cell proliferation, cell cycle, and cell survival using five different cancer cell lines, such as U-87, Panc-1a, SH-SY5Y, PC-3 and Hep3B, and one healthy cell line, HUVEC.

While garlic exosome therapy significantly reduced cancer cell viability, it had no cytotoxic effect on the normal cell line, HUVEC. Our data indicate that treatment of five cancer cell lines with garlic exosomes resulted in a significant rise in the expression of proapoptotic genes such as p53, Bax, Cas3, and Cas9, as well as a statistically significant decrease in the expression of the antiapoptotic gene Bcl-2. There are several mechanisms by which the p53 and pRb1 pathways can be inhibited in human cancers (Alshatwi et al., 2016). Garlic SEVs were shown to increase p53 expression while simultaneously decreasing Bcl-2 expression in five cancer cell lines. These findings suggest that garlic SEVs are involved in apoptosis as well as the antiproliferative action of garlic SEVs on cancer cell lines (Subash-Babu et al., 2017). Garlic SEVs have been shown to trigger apoptosis and enhance the expression of proapoptotic genes such as Cas3, Cas9, Bax, and p53, according to the data obtained in this study. Cas3, which is activated primarily by Cas9, is one of the most important executors of cellular apoptosis, and thus this finding suggests that the effect of garlic SEVs was not limited to abrogating cancer cell proliferation but also increased cell survival in normal cells, which is consistent with previous findings (Ouyang et al., 2012). In addition to the increase in apoptosis-related genes detected by PCR, parallel results observed by ELISA analyses revealed an increase in Cas3, which plays a key role in apoptosis (Figure 5). The TUNEL assay was also applied in order to detect apoptotic DNA fragmentation, which is commonly used to identify and quantify apoptotic cells, as well as to detect excessive DNA breakdown in cells (Loo, 2011). As quantifications and qualifications can be seen in Figure 6, fluorescence intensities were increased approximately 1.5-fold for U87, Panc-1a, Hep3b, PC-3, and SH-SY5Y cancer cell lines while this level was increased in healthy cell lines.

In order to investigate the connection between cell cycle arrest and cell death mechanisms, an annexin V/PI staining experiment was conducted for each cell line after treatment with garlic SEVs to determine the type of cell death mechanism. The apoptotic effect of garlic SEVs on certain types of cancer cell lines was indicated in recent research (Özkan et al., 2021). Similarly, in our research, apoptosis was observed at high levels in U-87, Hep3B, and Panc-1 cancer cell lines treated with 60 μg/mL garlic SEVs. Our findings revealed that the proportion of early and late apoptotic cells grew in U-87, Hep3B and Panc-1a cancer cell lines by approximately 73%, 82%, and 48%, respectively. However, necrosis was highly observed in SH-SY5Y cancer cell line. Although necrosis was observed in PC-3, both late and early apoptosis were higher with treatment with garlic SEV.

In conclusion, the evidence provided here clearly suggests that garlic SEVs have therapeutic potential on cancer cells while having no cytotoxic effect on the healthy cell line HUVEC. The apoptotic effect of garlic SEV on kidney and lung cancer cell lines has already been indicated in a recent study by Özkan et al. (2021). As a matter of fact, our findings revealed that these nanovesicles have an apoptotic effect on cancer cells too by increasing apoptotic Bax, p53, and Cas3 expression levels on both protein and mRNA levels (Özkan et al., 2021). While apoptosis was enhanced by garlic SEVs on cancer cells, there was an increase in some antiapoptotic Bcl-2 expression levels and a reduction in Cas3 activity on a healthy HUVEC cell line. This information confirms that SEV-like nanoparticles from garlic have the capacity to communicate across kingdom boundaries and may prove valuable in the development of novel anticancer therapies with minimal negative effects.

## 5. Conclusion

To summarize, plant-derived exosome-like nanovesicles such as garlic have great potential for therapeutic use, especially in the treatment of cancer. This is due to their inherent stability in plasma and their high concentration of bioactive lipids such as sphingolipids phospholipids glycolipids, proteins (HSPs, membrane transport proteins, signal transduction proteins antioxidant proteins), and nucleic acids. The study focused on extracting exosome-like nanovesicles from garlic and showed that these nanovesicles have a significant impact on reducing the viability of cancer cells from several cancer cell lines while leaving healthy cells unaffected. The nanovesicles also caused an upregulation of proapoptotic genes and a downregulation of antiapoptotic genes in cancer cells that were treated with them. This suggests that the nanovesicles play a role in inducing apoptosis and inhibiting the proliferation of cancer cells. Furthermore, our research indicated different types of cell death (apoptosis and necrosis) in certain cancer cell lines. This emphasizes the potential of garlic exosome-like nanovesicles as promising options for advanced and targeted anticancer treatments without negative side effects. This discovery opens up new possibilities for precision medicine in disease treatment.

## Abbreviations

SEV: small extracellular vesicles
HUVEC: human umbilical vein endothelial cell line
Hep3B: human hepatoma cell line
SH-SY5Y: human neuroblastoma cell line
Panc-1: human pancreatic adenocarcinoma cell line
U87: human glioblastoma cell line
PC-3: prostate cancer cell line
